# A Rare Cause of Life-Threatening Chest Pain: Kounis Syndrome

**DOI:** 10.3390/jcdd13020094

**Published:** 2026-02-14

**Authors:** Liangliang Jia, Yabin Liu, Xi Chen, Yufei Wang

**Affiliations:** 1 Department of Cardiology, The Second Affiliated Hospital, Zhejiang University School of Medicine, Hangzhou 310009, China; liuyabin0916@zju.edu.cn (Y.L.); 2508089@zju.edu.cn (X.C.); 22318109@zju.edu.cn (Y.W.); 2 State Key Laboratory of Transvascular Implantation Devices, Hangzhou 310009, China; 3 Heart Regeneration and Repair Key Laboratory of Zhejiang Province, Hangzhou 310009, China; 4 Transvascular Implantation Devices Research Institute, Hangzhou 310053, China

**Keywords:** Kounis syndrome, chest pain, allergic reaction, coronary vasospasm, acute coronary syndrome

## Abstract

Kounis syndrome, defined as an acute coronary syndrome triggered by allergic or hypersensitivity reactions, is a rare yet potentially life-threatening condition. This report details the case of a 50-year-old female patient presenting with recurrent chest pain, hypereosinophilia, and allergic comorbidities, who was ultimately diagnosed with type I Kounis syndrome. Modifications to her treatment regimen, including the administration of diltiazem and prednisolone, led to a complete resolution of her symptoms. This case highlights the critical importance of early diagnosis and timely intervention in managing Kounis syndrome. Early recognition is essential to prevent potentially fatal outcomes, thereby emphasizing the need for increased awareness among healthcare professionals.

## 1. Introduction

Kounis syndrome, alternatively termed allergic acute coronary syndrome (ACS) and initially described in 1991, encompasses a range of allergic reactions that can result in coronary vasospasm, plaque erosion, or stent thrombosis. Clinically, the syndrome is most commonly manifested by chest pain, which may be accompanied by other allergic symptoms such as urticaria or anaphylaxis. The diagnostic process relies heavily on clinical history, electrocardiographic alterations, and elevated cardiac biomarkers, necessitating a high degree of clinical suspicion due to its symptomatic overlap with more prevalent cardiac conditions [1,2]. Despite growing awareness, Kounis syndrome remains underdiagnosed, especially in patients who present with chest pain concurrent with mild or absent allergic symptoms [3]. In this context, we present a case of a 50-year-old female patient diagnosed with Kounis syndrome due to recurrent chest pain and easily neglected allergic reactions.

## 2. Case Report

**Figure 1:**
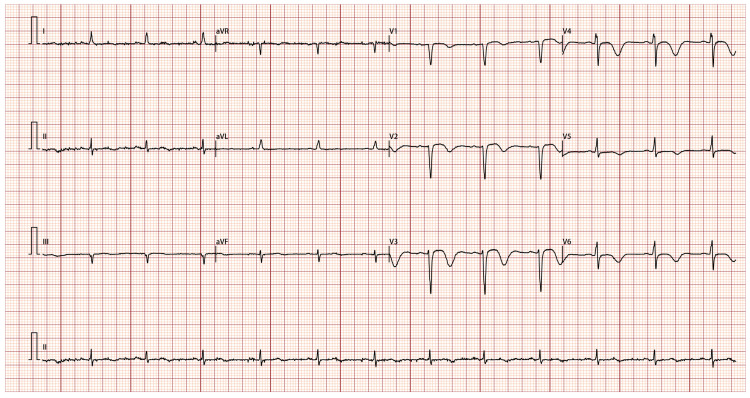
Electrocardiogram recorded at admission. Sinus tachycardia, V1, V2, V3, V4, V5 and V6 leads had ST-segment changes and T-wave inversions.

**Figure 2:**
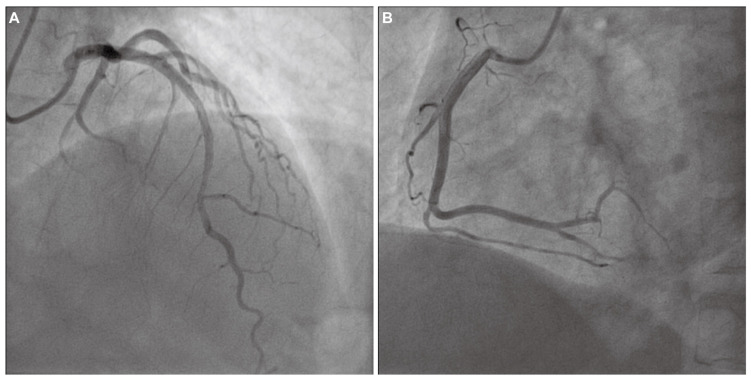
Coronary angiography. (**A**) Coronary angiography showed myocardial bridge in the mid-segments of left anterior descending artery, with a 50% luminal compression during the systolic phase. (**B**) Coronary angiography showed no remarkable findings on the right coronary artery.

A 50-year-old female patient was admitted to our hospital with a 20-day history of recurrent substernal chest pain radiating to the left shoulder and jaw. These episodes occurred daily at rest, lasted for several minutes, and resolved spontaneously. Prior to the initial onset of chest pain, the patient experienced intermittent episodes of upper abdominal pain and diarrhea for 10 days. During this period, she received anti-infective and gastric protective treatments at a local hospital, resulting in an improvement in her abdominal symptoms. The patient has a longstanding medical history of hypertension, bronchial asthma, allergic rhinitis, and multiple drug allergies, including hypersensitivity to lansoprazole, aspirin, latamoxef, and levofloxacin. She denied smoking and alcohol use. Her vital signs were a temperature of 36.5 °C, blood pressure of 110/82 mmHg, a respiration rate of 18 breaths per minute, and a pulse rate of 85 beats per minute. Cardiopulmonary examination was unremarkable. Abdominal and neurological examinations revealed no abnormalities. Her complete blood count showed an elevated absolute eosinophil count (1.1 × 10^9^/L) and eosinophil percentage (17.7%). Cardiac biomarkers showed elevated troponin-T (1.65 ng/mL) and brain natriuretic peptide (BNP, 307.5 pg/mL), but normal creatine kinase (CK)-MB. The electrocardiogram (ECG) at admission indicated sinus tachycardia, ST-segment changes and T-wave inversions in the anterior leads V1, V2, V3, V4, V5, and V6 (Figure 1). Echocardiography demonstrated regional wall motion abnormalities at the apex and inferior wall. She was initially diagnosed with non-ST-elevation myocardial infarction and then immediately sent for coronary angiography. The angiographic findings revealed a myocardial bridge in the mid-segments of the left anterior descending artery, exhibiting a 50% luminal compression during the systolic phase, without atherosclerotic stenosis (Figure 2A,B). Considering that the mild lesion of myocardial bridge cannot fully explain the repeated angina pectoris at rest in this patient, coronary vasospasm could not be ruled out. Due to the patient’s aspirin allergy, an alternative dual antiplatelet therapy regimen comprising cilostazol and clopidogrel was administered, in conjunction with the calcium channel blocker diltiazem, atorvastatin and pantoprazole. This prescription resulted in partial alleviation of cardiac symptoms. The patient was discharged in a stable condition after one week of observation.

**Figure 3:**
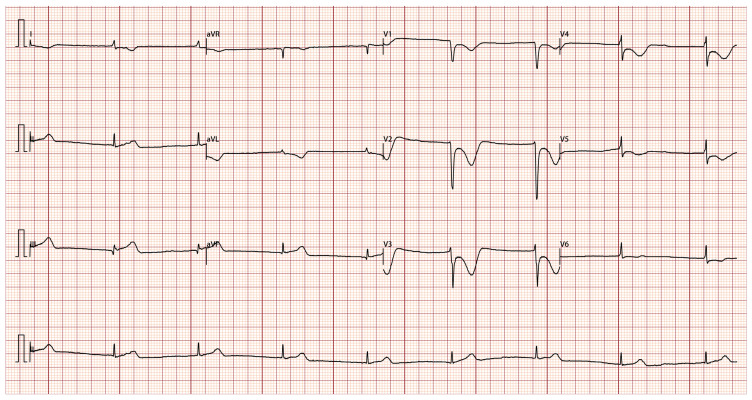
Electrocardiogram recorded during angina episode. Junctional escape rhythm, II, III, and aVF leads had elevated ST segments, V1, V2, V3, V4, V5 and V6 leads had depressed ST segments and T-wave inversions.

**Figure 4:**
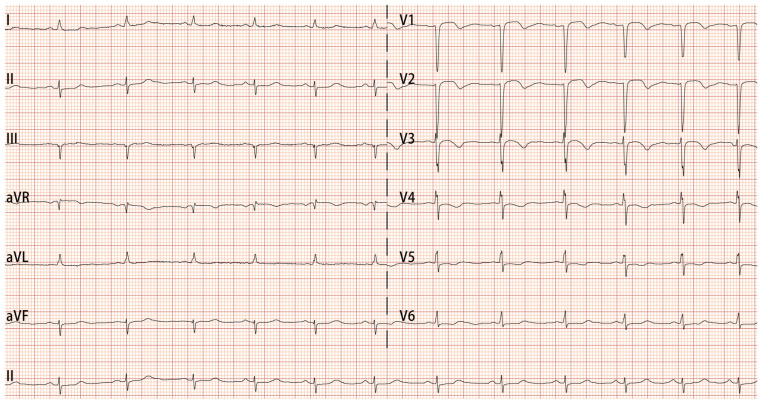
Electrocardiogram recorded during relieved angina episode. Sinus rhythm, V1, V2, V3, V4, V5 and V6 leads had ST-segment changes and T-wave inversions.

Despite adhering to the prescribed medication regimen after her initial discharge, the patient continued to experience recurrent chest pain and was readmitted to our hospital due to exacerbated episodes 2 months later. During the hospitalization, the patient experienced an acute onset of chest pain accompanied by dizziness and profuse sweating, which subsequently led to a brief episode of syncope lasting 1–2 min. ECG monitoring indicated junctional escape rhythm, with a heart rate of 40 beats per minute and blood pressure of 80/47 mmHg. An ECG was done at the attack and showed junctional escape rhythm, ST-segment elevation in the inferior leads II, III, and aVF, with reciprocal ST-segment depression and inverted T-wave changes in the anterior leads V1, V2, V3, V4, and V5 (Figure 3). Intravenous nitroglycerin provided a brief symptom relief, ECG was repeated and her ST-segment elevation was completely resolved (Figure 4). However, the patient continued to experience recurrent chest pain in the ensuing days of hospitalization. She was kept under cardiac monitors with serial ECG and troponin levels. Her cardiac biomarkers at admission showed elevated troponin-T (0.936 ng/mL), but normal CK-MB and BNP. Her hematological evaluation revealed persistent and worsening eosinophilia, with an absolute eosinophil count ranging from 1.1 to 6.83 × 10^9^/L and an eosinophil percentage between 17.7% and 64.4%. Additionally, serum immunoglobulin E (IgE) levels were found to be elevated at 234 IU/mL (reference range: <100 IU/mL). Bone marrow examination revealed no evidence of clonal hypereosinophilia (HE) or genetic mutations. The results of fecal parasite tests, antinuclear antibodies, antineutrophil cytoplasmic antibodies, and tumor biomarker assays were all negative. A gastrointestinal endoscopy conducted at her local hospital 3 months ago revealed bile reflux gastritis with erosion, but no signs of eosinophilic gastrointestinal disease. Consequently, secondary eosinophilia attributable to parasitic infections, connective tissue disorders, and neoplastic conditions can be largely excluded. Ultimately, based on the angiography, ECG, and elevated IgE levels, the patient was diagnosed with type I Kounis syndrome, characterized by an allergic reaction and coronary vasospasm, helping to rule out conditions like myocarditis or Takotsubo cardiomyopathy. Prednisolone (0.5 mg/kg/day) was timely initiated, while the therapeutic regimen comprising cilostazol, clopidogrel, diltiazem, and atorvastatin was continued, leading to complete resolution of chest pain. Given the patient’s known allergy to rabeprazole, pantoprazole, another proton pump inhibitor, was discontinued as it may have contributed to the chest pain. The patient was discharged from the hospital with a regimen of gradual steroid tapering. Six months after discharge, the patient successfully discontinued steroid therapy following the normalization of eosinophil levels.

## 3. Discussion

Current guidelines on ACS delineate various conditions in patients presenting with ACS symptoms, elevated troponin levels, and non-obstructive coronary arteries [4]. These conditions include coronary artery thrombosis, spasm, microvascular dysfunction, myocardial bridging, spontaneous coronary artery dissection and non-coronary causes such as myocarditis, Takotsubo cardiomyopathy, and hypersensitivity reactions. Kounis syndrome is a rare condition characterized by ACS precipitated by allergic reactions. A nationwide epidemiological study conducted in the United States indicated that the overall prevalence of Kounis syndrome among patients hospitalized for allergic and anaphylactic reactions was 1.1%, accompanied by an inpatient mortality rate of 7% [3]. Research indicates that the pathophysiology involves mast cell activation and the subsequent release of inflammatory mediators, leading to coronary vasospasm or plaque rupture [5]. Kounis syndrome manifests in various forms, including type I (with normal coronary arteries), type II (with pre-existing coronary artery disease), and type III (stent thrombosis or stent restenosis), which complicates the diagnostic process [5].

The extant literature suggests that Kounis syndrome is frequently underdiagnosed due to its clinical resemblance to more prevalent cardiac conditions [6]. The diagnosis of Kounis syndrome is primarily based on clinical presentation, laboratory findings, ECG, echocardiography, and coronary angiography [1]. In this case, diagnostic evaluation revealed elevated troponin levels and eosinophilia, increased IgE, and caused dynamic ECG changes, all of which corroborated the diagnosis of Kounis syndrome. It is worth noting that elevated tryptase levels may serve as a valuable biomarker for diagnosing anaphylaxis. Nevertheless, given the short half-life of tryptase, normal tryptase levels do not exclude the possibility of anaphylaxis or Kounis syndrome [7,8]. In addition, due to limited availability, tryptase testing was not performed in this case. Current literature indicates that the acute release of inflammatory mediators in Type I Kounis syndrome can induce coronary vasospasm without an increase in cardiac biomarkers (troponins or CK-MB) or can lead to coronary vasospasm culminating in acute myocardial infarction with elevated cardiac biomarkers [9]. In patients with type I Kounis syndrome, where electrocardiographic abnormalities are transient, it is crucial to conduct repeated ECG monitoring to facilitate the early and accurate identification of Kounis syndrome [9,10]. Ultimately, this case was diagnosed with Type I Kounis syndrome, as angiography did not reveal obstructive coronary artery disease. Currently, Kounis et al. propose that hypersensitivity or abnormal reactivity to an allergen should be encountered as potential causes of acute myocarditis. In this view, Kounis syndrome has been suggested as a multidisciplinary and multisystemic disorder [11]. Additionally, they propose that the combination of Kounis and Takotsubo-like syndromes may be present in specific cases [12]. Newer techniques, such as cardiac single photon emission computed tomography (SPECT) and cardiac magnetic resonance imaging (MRI), have demonstrated efficacy in detecting severe myocardial ischemia and differentiating Kounis syndrome from myocarditis or Takotsubo cardiomyopathy, particularly when coronary angiography appears normal in the type I variant of Kounis syndrome [6,7,13]. During this patient’s hospitalization, the utilization of cardiac SPECT and MRI could have substantially improved the diagnostic accuracy for Kounis syndrome. We acknowledge this as a limitation in the diagnostic process.

Due to the rarity of Kounis syndrome, proper management guidelines are still lacking. The management of Kounis syndrome depends on the specific type of the syndrome and necessitates the concurrent management of both allergic reactions and ACS [7]. Numerous medications have been documented as being associated with Kounis syndrome, including antibiotics, analgesics, antineoplastics, contrast media, proton pump inhibitors, anesthetics, and thrombolytics. Additionally, environmental exposures and various health conditions have been identified as potential precipitating factors [7]. Therefore, preventing future episodes is a crucial component of managing Kounis syndrome. Identifying and avoiding known triggers can decrease the probability of recurrence [7,9,14]. In the case we presented, the patient did not exhibit clearly identifiable allergic symptoms prior to the onset of chest pain; however, the deterioration of cardiac symptoms was associated with persistently elevated eosinophil and IgE levels. Consequently, we suspect that the patient’s chronic allergic conditions (e.g., asthma and allergic rhinitis) and drug hypersensitivity (e.g., antibiotics and proton pump inhibitors) are likely contributing factors to the development of Kounis syndrome. In cases of type I Kounis syndrome, the administration of antihistamines and corticosteroids is recommended to mitigate the allergic response. Moreover, the symptoms of coronary vasospasm can be effectively managed with vasodilators, such as nitroglycerin and calcium channel blockers, in hemodynamically stable patients [7,15]. In our case, the complete resolution of cardiac symptoms following therapy with prednisolone further supports the involvement of inflammation in Kounis syndrome and indicates corticosteroids can effectively mitigate allergic reactions and their associated cardiovascular consequences.

## 4. Conclusions

Kounis syndrome, characterized as an allergic ACS, represents a rare yet potentially fatal condition encountered in clinical practice. It is imperative for clinicians to maintain a heightened level of suspicion in patients presenting with recurrent angina and a history of allergies. Possibly due to the complexity of a disease process that involves both allergy and myocardial ischemia, guidelines in Kounis syndrome management are still lacking. Early corticosteroid therapy can avert catastrophic cardiac complications, highlighting the importance of tailored immunomodulatory strategies in this unique life-threatening disorder.

## Data Availability

The data presented in this study are available on request from the corresponding author due to reasons of sensitivity.

## Abbreviations

The following abbreviations are used in this manuscript:

ACS	Acute coronary syndrome
BNP	Brain natriuretic peptide
CK	Creatine kinase
ECG	Electrocardiogram
IgE	Immunoglobulin E
HE	Hypereosinophilia
SPECT	Single photon emission computed tomography
MRI	Magnetic resonance imaging
